# The Alien Plant Species Impact in Rice Crops in Northwestern Italy

**DOI:** 10.3390/plants12102012

**Published:** 2023-05-17

**Authors:** Ilda Vagge, Gemma Chiaffarelli

**Affiliations:** Department of Agricultural and Environmental Sciences, University of Milan, Via Celoria 2, I-20133 Milan, Italy

**Keywords:** biodiversity loss, alien invasion, alien flora, vegetation, weed species, phytosociology, organic farming, rice field, rice paddy

## Abstract

Alien species represent one of the causes of biodiversity loss, both in natural and anthropic environments. This study contributes to the assessment of alien species impact on Western Po Plain rice field cultivations, referring to different agricultural management practices and ecological traits. Flora and vegetation were studied (the latter through the phytosociological method), and α-biodiversity was estimated through Shannon and Simpson Indices. Results highlighted a significant floristic contingent depletion and increase in therophyte and alien components, compared to pre-existing studies (1950s); higher α-biodiversity levels in organic farms, compared to conventional farms, but also a higher invasive alien species percentage. The high deterioration of the territorial–landscape context appears to play a major role in shaping these patterns. Some of these alien species are particularly aggressive (e.g., *Murdannia keisak*), as confirmed by two experimental rice field plots which were left unharvested, continuously flooded, making it possible to assess the competitiveness between weed species. The detected weed vegetation is attributed to the *Oryzo sativae-Echinochloetum cruris-galli* association, already described for Southern Europe, with two different ecological and floristic variants. Future studies, by including other sites and framing their territorial–landscape context, may further complement this overview on the alien species distribution and behavior in rice fields, hence facilitating their strategic management.

## 1. Introduction

Alien species invasion is described as one of the causes of biodiversity loss by many scientific studies [1,2,3,4,5,6,7,8,9,10] and reports from world organizations such as the IUCN and UN [11,12,13,14,15]. This not only concerns natural and seminatural habitats, but also anthropic environments [3,9]. Biodiversity loss alters the structure of ecosystems, their functionalities, and leads to a decline in ecosystem services, economic losses and human health issues [16,17,18,19,20].

In Italy, 1628 alien plant taxa are documented (about 16% of total Italian flora) [21,22,23,24,25,26], and can be mainly found in anthropic settings, such as urban and agricultural areas, reforested areas and artificial water courses, where they often become the dominant component of vegetational associations. The Po Plain, a highly urbanized, industrialized and intensively cultivated lowland in Northern Italy, is the most affected Italian region, especially with respect to invasive alien species [9,21,26,27]. Here, alien species tend to constitute monospecific populations or to become predominant in some habitats. They significantly shape plant communities and threaten the autochthonous habitats, especially those of conservation interest [27,28]. Moreover, it has been shown that intensive, highly simplified, agricultural systems and anthropized territories represent a threat for the native flora while favouring alien species invasion [29]. Such territorial traits affect the vast majority of the Po Plain area, with oversized agricultural patches, often matched with anthropic linear infrastructures, not leaving room to natural and seminatural ecosystems.

For Italy, there are no recent specific studies on alien plant species impacts on irrigated crops (including rice crops), except for a study conducted in Sardinia [30], which only focused on floristic and not vegetational traits. The most recent updates on Italian rice fields weed alien species are Viggiani’s reports [31,32]: they provide a generalized overview on the occurring taxa in Italy, but they do not address any specific impact issue. Analysis of weed vegetation in rice fields in the Po Plain (the first Italian and European rice producer region [33]) dates back to more than 40 years ago [34,35,36]; updated and comprehensive floristic–vegetational studies focusing on alien species impacts (also in relation to biodiversity issues) are currently lacking.

In this work, we focused on agricultural settings related to rice cultivation in the Western Po Plain area. Here, the impact of alien species has been increasing over the years [9,37], mainly due to changes in rice cultivation techniques [31]. In Italy, until the early 1960s, rice cultivation was generally based on traditional systems (wet seeding with continuous rice paddies flooding, starting before sowing and ending a few weeks before harvest). Nowadays, traditional practices are uncommonly applied, being almost entirely replaced by rice cultivation systems based on simplified rotation or monoculture, dry seeding with postponed flooding or alternate wetting and drying systems [38,39,40,41]. Traditional cultivars are mostly replaced by short-stature rice varieties managed through conventional rice systems, involving deep soil tillage and the extensive application of chemical herbicides, pesticides and mineral fertilizers. These shifts in agronomic and water management techniques, together with the prolonged chemical weed control, led to changes in rice weed flora and fostered alien species invasion [31,32,37,38,42]. Rice paddy environments often constitute refuge areas for rare species, substituting the role of wetlands and protected habitats, which are currently severely threatened by climate change, changes in agricultural management practices as well as by alien species invasion [42,43,44,45,46,47].

Our study objectives were to assess the alien plant species occurrence and consistency among differently managed rice farming systems (conventional versus organic systems) and to estimate their impact on biodiversity. This study is part of a wider research project where agrobiodiversity traits are assessed trough multi-scale approaches, where the influence of landscape scale processes is also taken into account [48]. Floristic–vegetational studies are also being conducted on other spontaneous phytocoenoses types (such as other crop fields, field banks, field margins, uncultivated areas, hedgerows and tree lines, woody areas, ditches, wetlands). For landscape scale analyses, landscape ecology [49,50,51,52,53] and landscape bionomic [54,55] approaches are adopted, framing the ecological functional patterns influencing field scale biodiversity.

## 2. Materials and Methods

**Figure 1:**
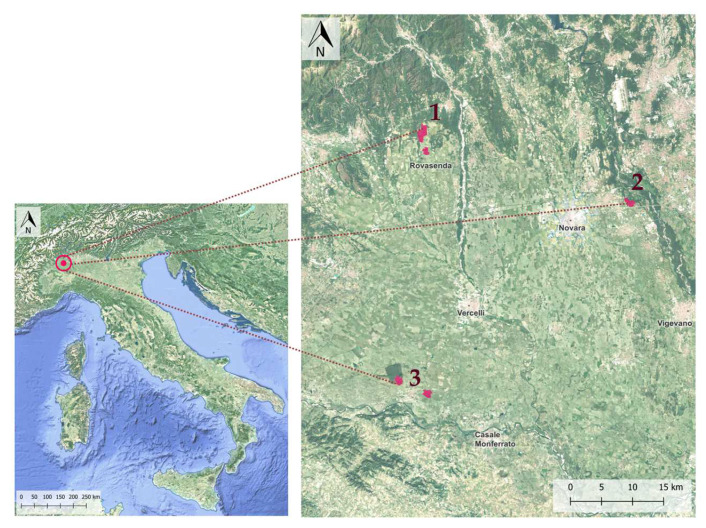
Study area location in the Western Po Plain region (north of Italy), and survey site location: site 1—Rovasenda, site 2—Romentino, site 3—Trino Vercellese (in red).

The study area is located in the Western Po Plain (Piedmont region) and consists of 3 sites belonging to the Vercelli and Novara districts (Rovasenda, Romentino and Trino Vercellese municipalities) (Figure 1). In each site, both organic and conventional farms were investigated.

**Figure 2:**
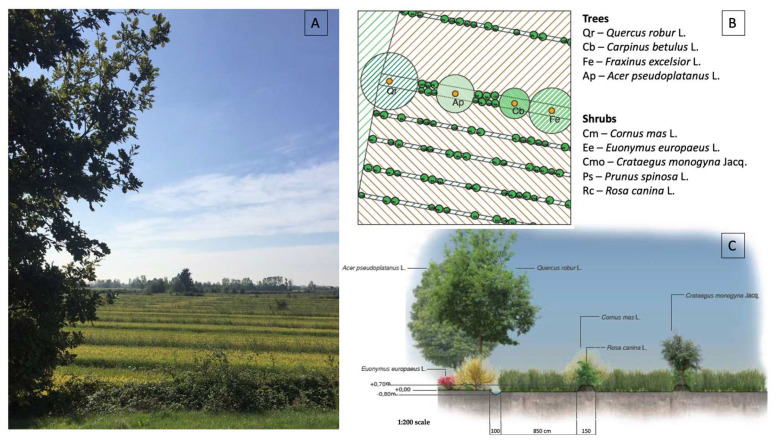
(**A**) A photo of rice fields of the organic farm Cascina dell’Angelo, Rovasenda, site 1; (**B**,**C**) an example of a planting scheme (plan (B); section (C)) of trees and shrubs in between fields and shrubs along in-field linear embankments, site 1.

The 3 sites were chosen because of the presence, among the organic farms, of 3 farms applying traditional cultivation practices based on crop diversification: rotations, continuous flooding conditions for rice fields, land races cultivation, presence of in-field linear embankments, maintenance of trenches along the field margins which are kept flooded throughout the year (allowing the persistence of diffused wet habitats for wild fauna and flora), spread hedgerows and tree lines in between fields (Figure 2).

The study area is characterized by a temperate bioclimate, ranging from temperate continental (from upper meso-temperate, upper sub-humid (site 3) to lower supra-temperate, lower humid (site 2)) to temperate oceanic (lower supra-temperate, lower humid (site 1)) according to the Rivas-Martinez *Worldwide Bioclimatic Classification System* [56,57,58,59,60].

Soils vary from young Entisols and Inceptisols (loamy texture; sub-acid (site 2) and calcareous (site 3) soils) to Riss alluvial terrace Alfisols (clay-silty texture, acid soils (site 1)) [61].

Flora and vegetation studies were carried out over 5 years (2018–2022) during the rice cultivation time period (from sowing to harvesting) among the 3 different sites, representing both conventional and organic rice cultivation systems (Figure 1). Only the species occurring inside the rice paddy were included in the analysis (i.e., excluding rice field banks, field edges and in-field embankments). Scientific nomenclature used is in accordance with the *Italian flora* system ([62,63,64,65]. We built a floristic list (see Table S1, Supplementary Materials) where each taxon was associated with its reference Raunkiær life form and chorotype [62,63,64,65,66]; ecological indicator values, according to the Ellenberg-Pignatti updated reference values, commonly adopted for the *Italian flora* [62,63,67,68,69]; and observation site. For life forms, the following abbreviations are applied: P = phanerophyte, Ch = chamaephyte, H = hemicryptophyte, G = geophyte, He/I = hydrophyte/helophyte, T = therophyte; scap = scapose, caesp = caespitose, bulb = bulbose, rhiz = rhizomatose, rept = reptant. The chorotypes of the native species were grouped into 7 biogeographical autochthonous regions: Boreal, Cosmopolitan, Euro-Asiatic, Orophyte South European, Mediterranean, Atlantic, Endemic. The others were classified as alien.

Floristic data were compared with Pomini’s pre-existing studies describing the rice fields weed flora detected in the same area (Vercelli and Novara districts) in the 1950s [70,71]. As in our study, Pomini’s data were collected during weed growing season (rice cultivation time frame). For data comparison, Pomini’s data were cleaned from species that were not detected within the rice paddy; scientific nomenclature was updated to current taxonomy; species were linked to their reference life forms and chorological traits, as made for current survey data. The floristic list obtained from Pomini’s studies also takes into account the previous study results [72,73,74]. Floristic studies following the 1950s have not been considered, because from the 1960s, agricultural practices widely changed (chemical synthesis products deployment) and the agri-environmental context became similar to that at present.

The vegetational study was based on the phytosociological method [75,76,77,78,79]: 40 phytosociological relevés were conducted among both conventional and organic farming systems, within the rice paddy, excluding rice field banks’ phytocoenoses. The relevés were tabulated, ordered and interpreted according to the phytosociological method, allowing us to identify vegetational communities with homogeneous floristic composition and ecology, which were then ascribed to the coherent syntaxonomic categories (associations, and the upper level syntaxa). Cover-abundance phytosociological indices (alpha-numerical Braun-Blanquet scale) were transformed into real numbers according to Van der Maarel [80] (conversion to central values, in order to compute the Specific Coverage Index (SCI) [78,81]). Statistical analyses were carried out using XLSTAT software, principal component analysis (PCA), and cluster analysis (using Euclidean distance as a dissimilarity coefficient). Syntaxa were classified according to the *Prodromo della vegetazione d’Italia* [82,83,84,85,86,87,88,89]. Quantitative biodiversity estimates were based on the J-Shannon Index [90] and E-Simpson Index [91], which were applied to phytosociological relevés. Four additional relevés were conducted over two years in two experimental plots (located in site 1) where rice was left unharvested and continuous flooding occurred during the whole year.

## 3. Results and Discussion

### 3.1. Flora

**Figure 3:**
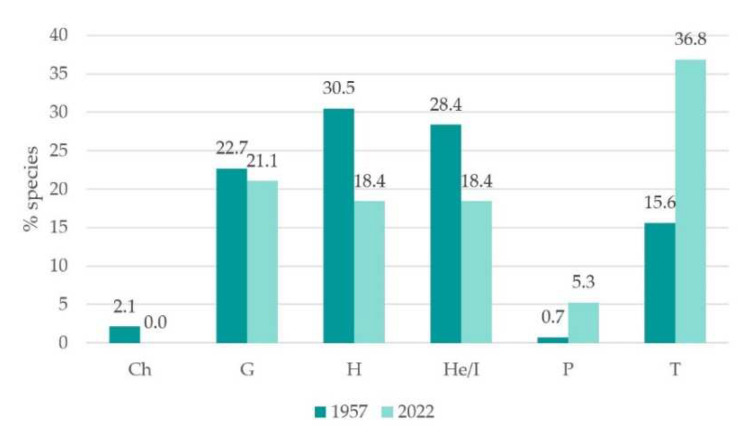
Life forms spectra (% total species) comparison 1950s versus current state. Abbreviations: Ch = chamaephyte, G = geophyte, H = hemicryptophyte, He/I = hydrophyte/helophyte, P = phanerophyte, T = therophyte.

Floristic analysis (see floristic list in Table S1, Supplementary Materials) detected 38 plant taxa inside the rice paddies. This is a significantly lower number if compared to previous censuses conducted in the Vercelli and Novara districts rice fields during the 1950s, where 141 taxa were detected [70]. The decrease in taxa number is linked to agricultural practice changes and chemical herbicide spread [31,32,37]. Indeed, current flora are dominated by short-cycle herbaceous species (therophytes: 36.84%), followed by geophytes (21.50%), hydrophytes/helophytes (18.42%) and perennial herbaceous species (hemicryptophytes: 18.42%) (Figure 3). In contrast, past flora was dominated by hemicryptophytes (30.5%) and hydrophytes/helophytes (28.37%), followed by geophytes (22.70%) and therophytes (15.60%) [70] (Figure 3). The current therophyte abundance is coherent with the influence of dry seeding, alternate wet and drying irrigation practices, and generally higher soil disturbance, which predominate. Similar patterns were detected in other diachronic studies on irrigated crops. For instance, Covarelli’s study compared corn weed flora historical data (1960s versus 2000s) and reported a significant increase in therophytes and a parallel decrease in hemicryptophytes, while chamephytes almost disappeared [37]. Although these results do not refer to rice crops, they highlight a general trend that can be related to the abandonment of traditional cultivation practices and a shift towards more intensive cultivation techniques.

**Figure 4:**
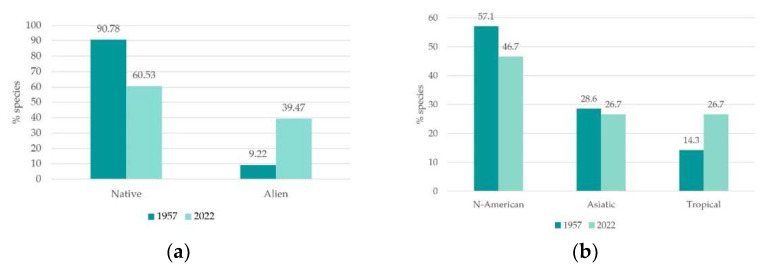
(**a**) Native and alien species proportion (% of total flora), 1950s versus current state floristic data comparison. (**b**) Alien species origin (% of total flora), 1950s versus current state floristic data comparison.

We observed a significant increase in alien species occurrence. Indeed, they represented 39.47% of total flora (of which 25.34% were invasive alien species), while they only represented 9.22% of total rice paddy flora in the 1950s [70] (Figure 4a). This result corresponds with the diachronic trends reported by other studies [9,37,92] and, accordingly to literature and to our study results, the occurrence of alien species poses a severe threat to biodiversity, as discussed hereafter. Compared to the 1950s, the alien species contingent has completely changed. The only invasive archaeophyte inventoried both in the 1950s and in current surveys was *Oryza sativa* L. var. *sylvatica* Chiappelli; the other 12 alien species observed in 1950s were not detected in the 3 study sites. The majority of the currently registered alien species are of northern origin: American (46.67%) followed by Asiatic (26.67%) and tropical (26.27%) (Figure 3b). Parallelly, in 1950s the species of American origin dominated (57.14%), followed by Asiatic (28.57%) and tropical (14.29) (Figure 4b). The decrease in North American species and the increase in the tropical ones might likely be a consequence of climate change and rice cultivation technique changes.

**Figure 5:**
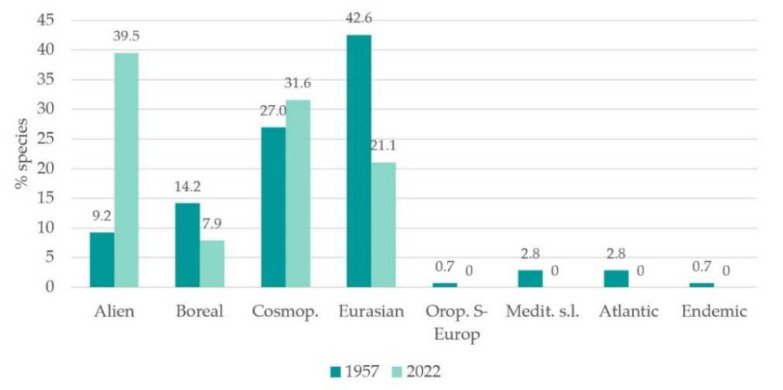
Floristic data: total chorotypes proportions (% of total flora) comparison (1950s versus current state). Abbreviations: Cosmop. = cosmopolitan; Orop. S- Europ. = orophyte South European; Medit. s.l. = Mediterranean.

From a chorological perspective, the autochthonous species contingent has decreased, if compared to the 1950s (Figure 5). Wide distribution species have increased, to the detriment of the Euro-Asiatic and Boreal ones. In the 1950s, a low occurrence of Mediterranean s.l. and Atlantic species was registered, as well as an endemic species (*Isoëtes malinverniana* Ces. & De Not.), which all disappeared in current investigations.

Most of current detected species are of wide distribution and trivial, with the exception of *Marsilea quadrifolia* L., which is classified as an endangered species by the IUCN [47,93]. Many species observed in the 1950s and are now missing, are classified by the IUCN as critically endangered species (*Isoëtes malinverniana* Ces. & De Not., *Pilularia globulifera* L.), endangered species (*Eleocharis carniolica* W.D.J.Koch, Carex buekii Wimm., *Typha minima* Funk ex Hoppe, *Sagittaria sagittifolia* L., *Myricaria germanica* (L.) Desv., *Utricularia vulgaris* L.), vulnerable species (*Salvinia natans* (L.) All.) and near threatened species (*Utricularia australis* R.Br., *Zannichellia palustris* L.) [3,93,94]. These species also have become very rare in natural wetland environments outside rice fields. These environments, due to anthropogenic and climatic causes, have been greatly reduced and are often polluted. The rice field environment, if properly managed, can serve as an important secondary habitat for the survival of threatened species.

### 3.2. Vegetation

**Figure 6:**
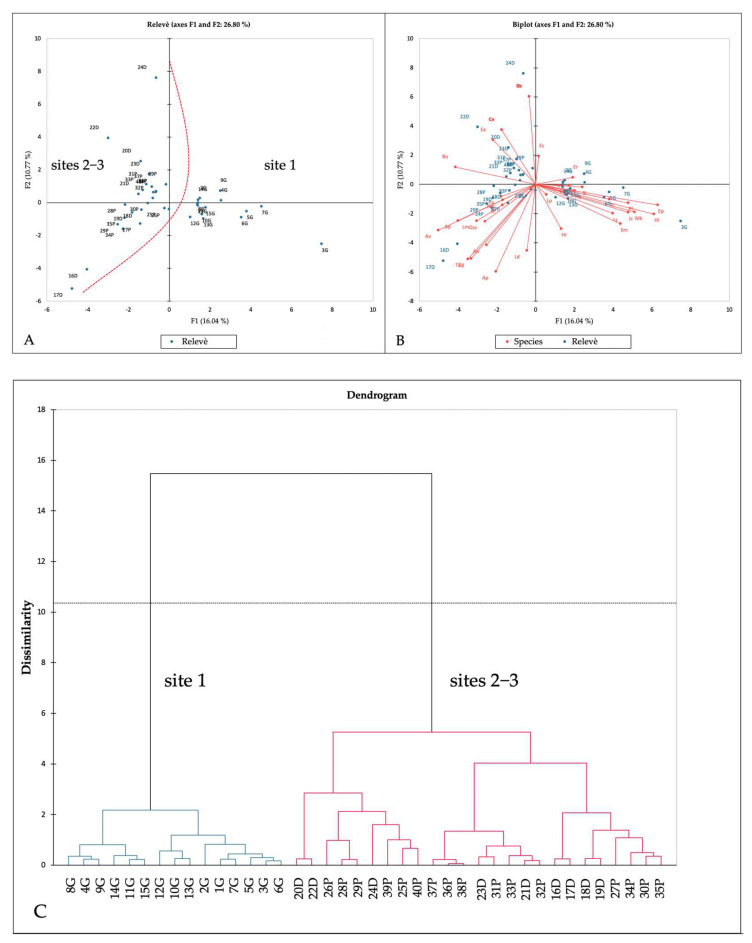
(**A**,**B**) PCA analysis results; (**C**) dendrogram (cluster analysis). See Table 1 for species and relevés code abbreviations.

Table 1 reports the phytosociological relevés, ordered and grouped (Table 1, see Appendix A for further details). Cluster analysis and PCA analysis highlighted two distinct groups (Figure 6). The first includes the relevés made at site 1, while the second one includes those made at sites 2–3. These two main clusters are differentiated by groups of species, mainly of alien origin (group 1: *Eleocharis flavescens* (Poir.) Urb., *Heteranthera limosa* (Sw.) Willd., *Murdannia keisak* (Hassk.) Hand.-Mazz. and *Juncus conglomeratus* L.; group 2: *Rotala densiflora* (Roth) Koehne, *Eleocharis acicularis* (L.) Roem. & Schult. And *Cyperus glomeratus* L.).

**Figure 7:**
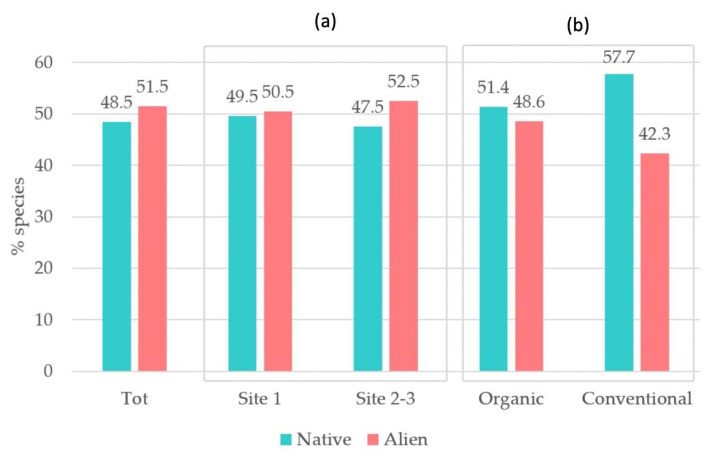
Native and alien species proportion (cover-abundance weighted % species). Comparison between: total vegetation; site 1 versus sites 2−3 (**a**); organic rice fields versus the conventional ones (**b**).

The vegetational study further highlighted alien species impact. Considering alien species coverage through SCI (Figure 7), alien species percentage overcame the native ones’ (51.5% versus 48.5%), especially in sites 2−3 (52.5% versus 47.5%). These impacts have to be linked to the higher anthropization degree of the territorial and landscape context of sites 2–3, which are located in the middle of the Po Plain. Sites 2 and 3 were more similar to each other than they were to site 1, from a biogeographical, floristic and ecological point of view.

Conventional rice fields showed a lower alien species percentage cover compared to the organic ones (Figure 7b). However, conventional fields generally had a lower occurrence and cover of the whole weed contingent: if we consider the mean species number, organic rice fields settled at 9.135, while the conventional ones settled at 5.778 (Table 2).

Periodical observations and surveys of the two experimental plots in site 1 (which were kept under continuous flooding conditions, leaving rice unharvested) showed an increase in alien species occurrence and coverage, to the detriment of the native ones (Table 3, see Appendix A for further details). Particularly, *Murdannia keisak* (Hassk.) Hand. −Mazz. exhibited greater aggressivity since it outcompeted native and non-native species and became dominant (cover values exceeding 75%). This led to a decrease of the overall species number (from 13 to 6) (Table 3). *Murdannia keisak* is a perennial geophyte which behaves as an annual species when growing in the rice field, where it easily reproduces via seeds. It also relies on vegetative propagation and tends to form dense and extensive populations, causing lodging of rice plants. These results seem to be in contrast with the results of a previous study conducted in Vercelli district [95], which specifically investigated *Murdannia keisak* responses to different water managements regimes. In this study, greenhouse experiments and field experiments indicated that continuous flooding conditions to be the best solution for preventing *Murdannia keisak* spread. Nonetheless, in this study, continuous flooding conditions were always paired to chemical pesticide application, while no pesticides were used in our experimental plots, as our aim was to simulate natural conditions which are likely to occur in wet natural habitats neighboring rice field habitats (acting as species source areas). These results could usefully orient targeted invasive alien species management, as well as other types of interventions aimed at supporting rice field system biodiversity (e.g., wetland restoration).

**Figure 8:**
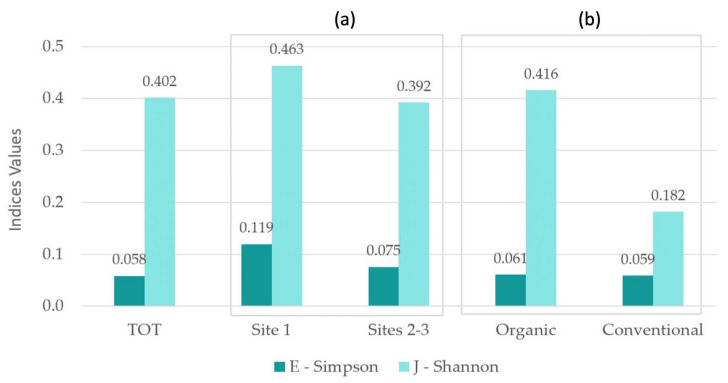
α-biodiversity calculated on vegetational data. Comparison between: total sites; site 1 and sites 2−3 (**a**); organic and conventional rice fields (**b**).

E-Simpson and J−Shannon indices where higher among organic rice fields (respectively, 0.061 versus 0.059 and 0.416 versus 0.182); the highest α-biodiversity values occurred in site 1 (respectively, 0.119 versus 0.075 in site 2−3 and 0.463 versus 0.392 in site 2−3), where the territorial and landscape context showed a higher naturality degree (Table 2; Figure 8).

Our study confirmed a significant alien species impact, even though it did not confirm a generalized direct negative relation between alien species and in-field biodiversity values. Alien species have a qualitative impact on biodiversity, more than the quantitative one. Our results support the following hypothesis: generally, the highly depleted ecological status of the territorial context entails higher vulnerability to the contamination of the floristic contingent by alien species [29,96]; higher biodiversity values can be linked to higher alien species occurrence (see organic field values) (Figure 7 and Figure 8). Where better territorial and landscape conditions occur (site 1), higher biodiversity values are linked to relatively lower alien species occurrence (site 1 versus site 2–3) (Figure 7 and Figure 8). All these results highlight the importance of adopting targeted precautions when addressing biodiversity issues in such depleted contexts. Notably, they highlight the need for multi-scale biodiversity assessment and management also through landscape-scale intervention strategies in order to successfully address biodiversity targets. These interpretations are aligned with Pellegrini’s study results (Northeastern Italy), which highlighted the role of landscape-scale extensive agricultural management in limiting alien plant invasion: even a small percentage of green infrastructure and extensive agricultural land amid the intensive one was proved to reduce alien species occurrence and facilitate native species diversity [29].

**Figure 9:**
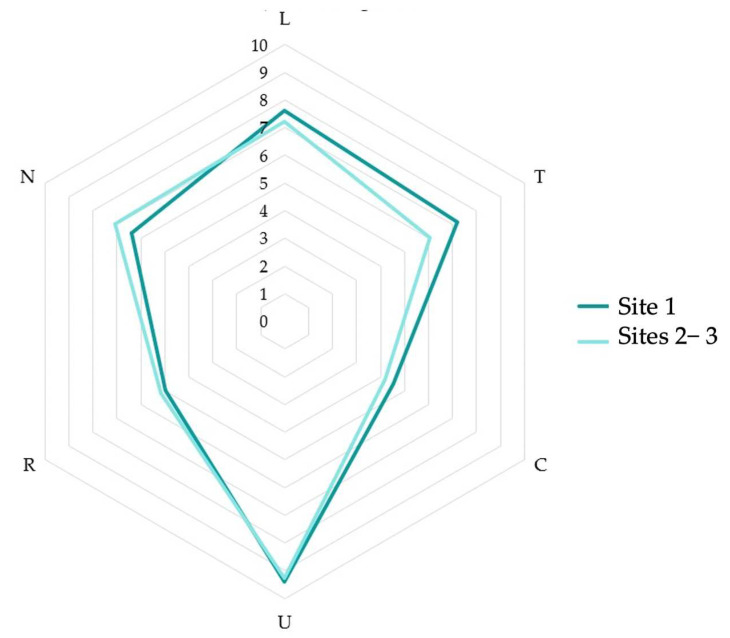
Ellenberg-Pignatti ecogram comparison (site 1 and sites 2–3), calculated on vegetational data.

The weed species contingent showed various differences between site 1 and sites 2–3 (see Figure 6, Table 1 and Table S1, Supplementary Materials). In site 1, the following species occurred, which were absent in sites 2–3: *Eleocharis flavescens* (Poir.) Urb., *Heteranthera limosa* (Sw.) Willd., *Murdannia keisak* (Hassk.) Hand.−Mazz. and *Juncus conglomeratus* L. In contrast, the following species only occurred in sites 2 and 3: *Rotala densiflora* (Roth) Koehne, *Eleocharis acicularis* (L.) Roem. & Schult. and *Cyperus glomeratus* L. These floristic differences are due to single species bio-evolutionary and distribution reasons, as well as to ecological reasons. In fact, Ellenberg−Pignatti ecograms [62,63,67,68,69] show comparable climatic conditions between site 1 and site 2–3 (except for slightly higher temperatures at site 1) (Figure 9). Nonetheless, they highlight soil differences: in site 1, soils appeared to be more acid and poorer in adsorbable nitrogen content (Figure 9). Therefore, different soil characteristics may explain the different distribution of weed species in rice fields.

There are no recent phytosociological studies of Italian rice paddy vegetation and its syntaxonomic characterization.The most recent are those related to the Pavia and Vercelli districts [34,35] and Ferrara district [36]. The phytosociological data reported in these studies were used in a study by Carretero [97] concerning European rice field vegetation and reviewing the *Oryzetea sativae* Miyawaki 1960 class.

The vegetation detected in the study area (Table 1, Figure 6) can be ascribed to the *Oryzo sativae*−*Echinochloetum cruris*−*galli* Soo 1946 ex Ubrizsy 1948 association (an European widely distributed syntaxa [81]), even if depleted; indeed, only *Echinochloa crus*−*galli* (L.) P. Beauv. occasionally occurs among the overall character-species set. The association belongs to the *Oryzo*−*Echinochloion oryzoidis* O. Bolos & Masclans 1955 alliance, *Cypero*−*Echinochloetalia oryzoidis* O. Bolos & Masclans 1955 order, *Oryzetea sativae* Miyawaki 1960 class; this class includes weed vegetation of rice fields comprising vascular phanerogams and cryptogams, particularly algae. This vegetation might be referred to the thermophilic *paspaletosum distichi* W. Koch 1954 ex Carretero 1989 sub-association, which was identified for the Iberian Peninsula, Southern France and Italy [97], even though only *Lindernia dubia* (L.) Pennell was detected among the sub-association differential-species set. A variant with *Eleocharis flavescens* (Poir.) Urb., *Heteranthera limosa* (Sw.) Willd., *Murdannia keisak* (Hassk.) Hand.−Mazz. is identified at site 1, indicating more acidic substrates poor in organic matter (Table 1, Figure 6).

In addition to the characteristic and differential species of the association and the upper syntaxa, companion species are listed in Table 1. Among companion species, we underline the occurrence of species typical of marshy environments ascribed to the *Phragmito australis*−*Magnocaricetea elatae* Klika in Klika & Novk 1941 class (such as: *Typha latifolia* L., *T. angustifolia* L., *Alisma plantago-aquatica* L.), that come from the neighboring wet environments (channels, irrigation ditches and ponds). Among the companion species, there were also nitrophilous, hygrophilous pioneer species belonging to the *Bidentetea tripartitae* Tuxen, Lohmeyer & Preising ex Von Rochow 1951 class (such as: *Bidens frondosa* L. and *Persicaria lapathifolia* (L.) Delarbre), that come from the dry environments (field banks, in-field embankments, uncultivated areas).

Further Northwestern Po Plain data collection is needed in order to build a more comprehensive overview of rice paddies’ floristic and vegetational traits, allowing description of new sub-associations able to demonstrate the floristic and ecological characteristics of the rice fields belonging to these Po Plain districts.

## 4. Conclusions

This study contributes in updating the current knowledge on rice fields’ weed flora and vegetation in Vercelli and Novara Western Po Plain districts. Specifically, it depicts a significant change in rice paddies’ spontaneous weed flora, compared to the 1950s: the overall taxa number has dramatically decreased; therophyte species, tightly linked to higher variability and disturbance of soil conditions, are favored by current cultivation practices, and have become predominant; current flora is depleted also from a chorological point of view, and the alien species proportion has significantly increased. Moreover, species of conservation interest which were detected in the 1950s were not encountered (except for *Marsilea quadrifolia* L.). This shows how the current rice field environmental and management conditions in the Western Po Plain are undermining the capability of rice field systems to behave as spread wet habitats supporting agrobiodiversity.

Moreover, vegetational studies have shown how organic rice field management sustains higher α-biodiversity values, compared to conventional techniques. Nonetheless, organic farms’ rice field phytocoenoses also host higher invasive alien species amounts. The results suggested that the depleted, overexploited territorial and landscape context might play a major role in enhancing the vulnerability to alien species invasion (differences between site 1 and sites 2–3, located in the middle of the intensively cultivated Po Plain). *Murdannia keisak* (Hassk.) Hand.−Mazz. appeared to be the most aggressive alien species among permanently flooded areas (such as wetlands), at site 1. Finally, vegetation studies showed how the distribution of rice field weed species (especially alien species) is mainly related to edaphic parameters, especially soil pH and organic matter content. In fact, a variant of the *Oryzo sativae*−*Echinochloetum cruris*−*galli* Soo 1946 ex Ubrizsy 1948 association was identified in site 1, and was related to more acidic and organic matter-poor soils.

Given the recognized growing impacts of alien species invasion, the here-detected patterns between biodiversity values, alien species occurrence, and territorial–landscape context deterioration, urge further floristic–vegetational investigations of Po Plain rice fields, also through multi-scale biodiversity assessment approaches. This could help in identifying targeted precautions for addressing biodiversity issues in Po Plain rice lands. Rice fields, if properly managed, are wet habitats that can act as biodiversity source areas and plant diversity conservation sites, where productive and conservation functions can positively coexist [42,46,47]. Rice fields are also pivotal habitats for faunal conservation: indeed, rice lands often host high conservation interest areas, such as “Natura 2000” European Network sites [98,99]. However, for effective agrobiodiversity support, the influence of the upper scale landscape context should be primarily taken into account [100,101,102]. Indeed, landscape system ecological rehabilitation and diversification can directly and indirectly influence rice field and, more generally, crop field biodiversity and alien species invasion patterns [29,96,100,101,102,103,104,105]. Hence, multi-scale biodiversity assessments are needed for orienting landscape scale management strategies, in order to successfully address biodiversity targets [106]. Our study results would benefit from the integration of landscape scale ecological analyses as well as from the assessment of the out-field spontaneous flora and vegetation, allowing better framing and understanding of the reasons behind the alien species impact patterns.

## Figures and Tables

**Table 1:** Phytosociological table reporting the total 40 relevés (for details regarding date, locality, organic/conventional farm and sporadic species, see Appendix A).

		SITE 1 (G)															SITES 2 (D)−3 (P)																										
		8G	4G	9G	14G	11G	15G	12G	10G	13G	2G	1G	3G	7G	5G	6G	20D	22D	26P	28P	29P	24D	39P	25P	40P	37P	36P	38P	23D	31P	33P	21D	32P	16D	17D	18D	19D	27P	34P	30P	35P		
Coverage (%)		95	100	100	100	100	100	100	100	100	100	100	100	100	100	100	100	95	100	100	100	100	100	100	100	100	100	100	95	100	100	100	100	100	100	100	100	100	100	100	100	pres.	freq.
Area (m^2^)		70	80	70	85	100	90	90	75	90	90	90	80	70	60	80	100	100	80	90	90	100	100	90	100	100	90	95	100	95	85	100	90	100	100	100	100	80	90	90	100		
Species number		8	10	7	10	7	10	9	10	10	7	9	12	8	8	9	9	9	8	9	11	5	4	9	7	4	5	4	3	8	7	7	9	11	14	10	10	10	9	7	8		
Os *Oryza sativa* s.l.		5.5	5.5	5.5	5.5	5.5	5.5	5.5	5.5	5.5	4.5	5.5	5.5	5.5	5.5	5.5	5.5	4.4	5.5	5.5	5.5	5.5	5.5	5.5	5.5	5.5	5.5	5.5	5.5	5.5	5.5	5.5	5.5	4.4	5.5	5.5	5.5	5.5	5.5	5.5	5.5	40	V
**Char. and diff. species of *Oryzo sativae−Echinocletum cruris galli***																																											
Ec *Echinocloa crus−galli* (L.) P. Beauv.		1.1	+	1.2	+0.2	1.2	+	−	+0.2	1.1	+	1.2	+	+0.2	1.2	−	−	2.3	2.2	1.2	+	1.1	+	1.2	+	+	+	−	−	+0.2	+	+	+	−	+0.2	+0.2	1.2	+	+	1.2	+	34	V
Ld *Lindernia dubia* (L.) Pennell		−	−	−	−	−	−	+	+	+	−	+	−	−	1.1	+	−	−	−	−	−	−	−	−	−	−	−	−	−	−	−	−	−	2.2	+	+	+	−	−	−	−	10	II
**Char. and diff. species of the upper units (*Oryzo−Echinochloion oryzoidis* alliance, *Cypero−Echinochloetalia oryzoidis* order, *Oryzetea sativae* class)**																																											
Sm	*Schoenoplectiella mucronata* (L.) J. Jung & H.K. Choi	2.3	3.4	2.3	1.2	1.2	2.2	1.1	1.2	1.1	3.3	+.2	2.3	3.3	2.3	3.4	+	1.2	3.3	+	+0.2	−	−	2.2	−	−	1.1	+	−	+	+	−	+0.2	+	1.1	−	+	+.2	1.2	1.1	2.2	33	IV
Hr	*Heteranthera reniformis* Ruiz & Pav.	−	1.2	−	1.2	3.3	2.2	2.3	2.4	3.3	1.1	+	2.3	−	−	−	−	−	−	−	+	−	−	1.1	−	−	−	−	−	+	−	3.3	+	−	+	+	+	2.3	2.3	1.2	1.3	22	III
Av	*Ammania verticillata* (Ard.) Lam.	−	−	−	+	−	−	−	−	−	−	−	−	−	−	−	+	+	+	−	−	−	−	1.1	−	+	1.2	+	−	+	3.3	2.3	1.2	2.2	4.4	1.2	2.3	2.2	2.3	2.2	3.3	20	III
Ep	*Eleocharis flavescens* (Poir.) Urb.	+0.2	1.2	+	+0.2	1.3	1.2	−	+0.2	1.2	1.1	+0.2	1.2	2.3	3.4	2.4	−	−	−	−	−	−	−	−	−	−	−	−	−	−	−	−	−	−	−	−	−	−	−	−	−	14	II
Hl	*Heteranthera limosa* (Sw.) Willd.	+	+	−	+0.2	−	+	1.2	1.3	−	−	−	3.4	2.3	3.4	1.2	−	−	−	−	−	−	−	−	−	−	−	−	−	−	−	−	−	−	−	−	−	−	−	−	−	10	II
Mk	Murdannia keisak (Hassk.) Hand.−Mazz.	−	+	−	1.1	−	+	−	+	−	+0.2	+	3.4	−	−	+	−	−	−	−	−	−	−	−	−	−	−	−	−	−	−	−	−	−	−	−	−	−	−	−	−	8	I
Rd	*Rotala densiflora* (Roth) Koehne	−	−	−	−	−	−	−	−	−	−	−	−	−	−	−	−	−	−	−	−	−	−	−	−	+	+	+	−	+	1.1	−	+	+	1.2	+	1.2	−	1.1	1.1	2.3	13	II
Ea	*Eleocharis acicularis* (L.) Roem. & Schult.	−	−	−	−	−	−	−	−	−	−	−	−	−	−	−	+	+.2	−	−	−	−	−	−	−	−	−	−	1.2	+	1.2	1.2	−	−	−	+	−	−	−	−	−	7	I
Oss	*Oryza sativa* L. var. *sylvatica* Chiappelli	−	−	−	−	−	−	−	−	−	−	−	−	−	−	−	−	−	−	1.2	2.2	−	−	+0.2	−	−	−	−	−	−	−	−	−	−	+0.2	−	−	+0.2	−	−	−	5	I
**Others species**																																											
Pl	*Persicaria lapathifolia* (L.) Delarbre	2.3	+	1.1	−	2.2	−	1.1	+	+0.2	+	2.3	2.2	1.2	+	2.3	1.1	−	1.1	−	+	−	+	1.2	+	−	−	−	−	−	1.2	−	1.2	−	−	+	−	+	−	−	−	23	III
Ap	Alisma plantago−aquatica L.	1.1	−	−	−	1.1	+	+	1.2	1.1	−	+0.2	1.2	−	−	−	−	−	1.1	+	+0.2	−	−	+	−	−	−	−	−	−	−	1.2	+	4.4	2.3	2.2	1.2	+0.2	+	+0.2	−	21	III
Bf	*Bidens frondosa* L.	+	+	−	+	−	+	+	−	+	−	−	−	−	−	+	+	+	+	2.2	−	−	+	−	+	−	−	−	−	−	−	−	−	+	+	+	1.2	−	−	−	+	18	III
Bu	*Butomus umbellatus* L.	−	−	−	−	−	−	−	−	−	−	−	−	−	−	−	1.2	3.2	−	1.2	1.2	−	−	−	−	−	−	−	+	+	−	+0.2	1.2	1.1	+	−	+	−	−	−	−	11	II
Lm	*Lemna minor* L.	−	−	−	−	−	−	−	−	−	−	−	−	−	−	−	−	−	−	+0.2	2.3	−	−	+0.3	1.1	−	−	−	−	−	−	−	−	+	−	−	−	+	1.1	−	+	8	I
Ta	*Typha angustifolia* L.	−	−	−	−	−	−	−	−	−	−	−	−	−	−	−	−	−	−	−	−	−	−	−	−	−	−	−	−	−	−	−	−	1.2	+	−	−	+	+	−	−	4	I
Jc	*Juncus conglomeratus* L.	−	−	−	−	−	−	−	−	1.2	−	−	1.1	1.1	−	+	−	−	−	−	−	−	−	−	−	−	−	−	−	−	−	−	−	−	−	−	−	−	−	−	−	4	I
Cg	*Cyperus glomeratus* L.	−	−	−	−	−	−	−	−	−	−	−	−	−	−	−	−	−	+	−	+	−	−	−	−	−	−	−	−	−	−	−	−	+0.2	2.3	−	−	−	−	−	−	4	I
Mq	*Marsilea quadrifolia* L.	−	−	−	−	−	−	−	−	−	−	−	−	−	−	−	−	−	−	−	−	−	−	−	+	−	−	−	−	−	−	−	−	−	−	−	−	−	1.2	−	2.3	3	I
Tl	*Typha latifolia* L.	−	−	+	−	−	−	−	−	−	−	−	+	−	−	−	−	−	−	−	−	−	−	−	−	−	−	−	−	−	−	−	−	−	−	−	−	−	−	−	−	2	I
Er	*Elymus repens* (L.) Gould	−	+	+0.2	−	−	−	−	−	−	−	−	−	−	−	−	−	−	−	−	−	−	−	−	−	−	−	−	−	−	−	−	−	−	−	−	−	−	−	−	−	2	I
Vb	*Veronica beccabunga* L.	−	−	−	+	−	+	−	−	−	−	−	−	−	−	−	−	−	−	−	−	−	−	−	−	−	−	−	−	−	−	−	−	−	−	−	−	−	−	−	−	2	I
Cs	*Cyperus strigosus* L.	−	−	−	−	−	−	−	−	−	−	−	−	−	−	−	+	1.2	−	−	−	−	−	−	−	−	−	−	−	−	−	−	−	−	−	−	−	−	−	−	−	2	I
Ce	*Calamagrostis epigejos* (L.) Roth	−	−	−	−	−	−	−	−	−	−	−	−	−	−	−	+	1.2	−	−	−	−	−	−	−	−	−	−	−	−	−	−	−	−	−	−	−	−	−	−	−	2	I
Pa	*Phragmites australis* (Cav.) Trin. ex Steud.	−	−	−	−	−	−	−	−	−	−	−	−	−	−	−	−	−	−	+	+	−	−	−	−	−	−	−	−	−	−	−	−	−	−	−	−	−	−	−	−	2	I
**Sporadic species**		−	−	−	−	−	−	1	−	−	−	−	1	1	1	−	−	−	−	−	−	3	−	−	1	−	−	−	−	−	−	−	−	−	1	−	−	−	−	−	−		

**Table 2:** α-biodiversity calculated on vegetational data and average number of taxa. Comparison between: total sites; site 1 and sites 2−3; organic and conventional rice fields.

	TOT	Site 1	Sites 2–3	Organic	Conventional
E—Simpson	0.058	0.119	0.075	0.061	0.059
J—Shannon	0.402	0.463	0.392	0.416	0.182
Average taxa number	8.275	8.933	7.880	9.135	5.778

**Table 3:** Phytosociological table reporting the 4 relevés related to the 2 experimental plots (site 1) (see Appendix A for further details).

	1	2	3	4
Coverage (%)	100	100	100	100
Area (m^2^)	90	80	80	80
Species number	13	10	7	6
*Oryza sativa* s.l.	3.3	2.3	1.1	+
*Murdannia keisak* (Hassk.) Hand.−Mazz.	4.5	4.4	5.5	5.5
*Schoenoplectiella mucronata* (L.) J. Jung & H.K. Choi	2.3	3.4	1.1	1.1
*Heteranthera reniformis* Ruiz & Pav.	3.3	1.2	+0.2	1.2
*Eleocharis flavescens* (Poir.) Urb.	1.2	2.2	−	−
*Echinocloa crus−galli* (L.) P. Beauv.	−	+0.2	−	+0.2
*Persicaria lapathifolia* (L.) Delarbre	1.1	1.1	+	−
*Alisma plantago−aquatica* L.	1.1	1.2	−	−
*Bidens frondosa* L.	1.2	+	+	−
*Typha angustifolia* L.	+	−	+	1.1
*Typha latifolia* L.	1.1	+	−	−
*Polygonum aviculare* L.	1.1	−	−	−
*Robinia pseudoacacia* L.	(+)	−	−	−
*Salix alba* L.	(+)	−	−	−

## Data Availability

The data presented in this study are available on request from the corresponding author.

## Supplementary Materials

The following supporting information can be downloaded at: https://www.mdpi.com/article/10.3390/plants12102012/s1, Table S1: Floristic List.

## Appendix A

**Table 1:** Localities and dates: rel. 1G, 2G Organic Farm Cascina dell’Angelo, Rovasenda (VC) 8 September 2019; rel. 3G, 4G, 5G, 6G, 7G, 8G, 9G Organic Farm Baraggia, Rovasenda (VC) 24 July 2018; rel. 10G Organic Farm Baraggia, Rovasenda (VC) 14 July 2022; rel. 11G, 12G, 13G Organic Farm Cascina Teglio, Rovasenda (VC) 23 July 2019; rel. 14G, 15G Conventional Farm Rovasenda (VC) 14 July 2022; rel. 16D, 17D Organic Farm Dulcamara, Romentino (NO) 23 July 2019; rel. 18D, 19D Organic Farm Dulcamara Romentino (NO) 15 September 2020; rel. 20D, 21D, 22D Organic Farm Romentino (NO) 6 June 2020; rel. 23D, 24D Conventional Farm Santuario del Varallino, Romentino (NO) 6 June 2020; rel. 25P, 26P, 27P, 28P, 29P Organic Farm Priorato, Trino Vercellese (VC) 26 July 2019; rel. 30P, 31P, 32P, 33P, 34P, 35P Organic Farm Priorato, Trino Vercellese (VC) 14 July 2022; rel. 36P, 37P, 38P Conventional Trino Vercellese (VC) 14 July 2022; rel. 39P, 40P Conventional Farm Trino Vercellese (VC) 26 July 2019.

Sporadic species—rel. 3G: *Lythrum salicaria* L. +, rel. 5G: *Hypericum perforatum* L. +, rel. 7G: *Setaria italica* subsp. *viridis* (L.) Thell. +, rel. 12G: *Lolium perenne* L. +, rel. 17D: *Artemisia vulgaris* L. +; rel. 24D: *Commelina communis* L. +, *Erigeron canadensis* L. +, *Amaranthus retroflexus* L. 2.3, rel. 40P: *Portulaca nitida* (Danin & H.G. Baker) Ricceri & Arrigoni +.

**Table 3:** Locality and dates: Organic Farm Cascina dell’Angelo, Rovasenda (VC), rel. 1: 13 July 2018, 2: 24 July 2018, rel. 3, 4: 23 July 2019.
