# Supplementary material for: The Alien Plant Species Impact in Rice Crops in Northwestern Italy
Source: Plants (Basel). 2023 May 17;12(10):2012. doi: 10.3390/plants12102012 (PMC10223007; doi:10.3390/plants12102012)
Supplement: Supplementary file 1 [file plants-12-02012-s001.zip › plants-2326323-supplementary.pdf]

Supplementary materials

Table S1. Floristic list. Abbreviations: Ch = chamaephyte, G = geophyte, H = hemicryptophyte, I = hydrophyte; He = helophyte, P = phanerophyte, T = therophyte, bulb = bulbose, caesp = caespitose, nat = floating, rad = rooted, rept = reptant, rhiz = rhizomatose, scap = scapose.

Taxon	Life form	Chorotype	Exoticity	IUCN (Italy)	Site 1	Site 2	Site 3
Alisma plantago-aquatica L.	I rad	Sub-Cosmopolitan	Native		X	X	X
Amaranthus retroflexus L.	T scap	N-American	Invasive neophyte			X	
Ammania verticillata (Ard.) Lam.	T scap	W-Asiatic	Random neophyte		X	X	X
Artemisia vulgaris L.	H scap	Euro-Asiatic	Native			X	
Bidens frondosa L.	T scap	N-American	Invasive neophyte		X	X	X
Butomus umbellatus L.	I rad	Euro-Asiatic	Native			X	X
Calamagrostis epigejos (L.) Roth	H caesp	Sibero-European	Native			X	
Commelina communis L.	G bulb	E-Asiatic	Invasive neophyte			X	
Cyperus glomeratus L.	T scap	Paleo-Subtropical	Native			X	X
Cyperus strigosus L.	H caesp	N-American	Naturalized neophyte			X	
Echinocloa crus-galli (L.) P. Beauv.	T scap	Sub-Cosmopolitan	Native		X	X	X
Eleocharis acicularis (L.) Roem. & Schult.	G rhiz	Sub-Cosmopolitan	Native			X	X
Eleocharis flavescens (Poir.) Urb.	T scap	N-American	Invasive neophyte		X		
Elymus repens (L.) Gould	G rhiz	Circumboreal	Native		X		
Erigeron canadensis L.	T scap	N-American	Invasive neophyte		X	X	
Heteranthera limosa (Sw.) Willd.	I rad	American	Random neophyte		X		
Heteranthera reniformis Ruiz & Pav.	I rad	Neotropical	Invasive neophyte		X	X	X
Hypericum perforatum L.	H scap	Cosmopolitan	Native		X		
Juncus conglomeratus L.	G rhiz	Sibero-European	Native		X		
Lemna minor L.	I nat	Sub-Cosmopolitan	Native			X	X
Lindernia dubia (L.) Pennell	T scap	N-American	Invasive neophyte		X	X	
Lolium perenne L.	H caesp	Circumboreal	Native		X		
Lythrum salicaria L.	H scap	Sub-Cosmopolitan	Native		X		
Marsilea quadrifolia L.	I rad	Euro-Asiatic	Native	EN			X
Murdannia keisak (Hassk.) Hand.-Mazz.	G bulb	Asiatic	Invasive neophyte		X		
Oryza sativa L. var. *sylvatica* Chiappelli	T scap	Asiatic	Invasive archeophyte			X	X
Persicaria lapathifolia (L.) Delarbre	T scap	Cosmopolitan	Native		X	X	X
Phragmites australis (Cav.) Trin. ex Steud.	G rhiz	Sub-Cosmopolitan	Native				X
Polygonum aviculare L.	T rept	Cosmopolitan	Native		X		
Portulaca oleracea L.	T scap	Paleotropical	Cryptogenic				X
Robinia pseudoacacia L.	P caesp	N-American	Invasive neophyte		X		
Rotala densiflora (Roth) Koehne	T scap	Paleotropical	Naturalized neophyte			X	X
Salix alba L. (pl.)	P scap	Paleotemperate	Native		X		
Schoenoplectiella mucronata (L.) J. Jung & H.K. Choi	He	Cosmopolitan	Native		X	X	X
Setaria italica subsp. *viridis* (L.) Thell.	T scap	Sub-Cosmopolitan	Native		X		
Typha angustifolia L.	G rhiz	Circumboreal	Native			X	X
Typha latifolia L.	G rhiz	Cosmopolitan	Native		X		
Veronica beccabunga L.	H rept	Euro-Asiatic	Native		X		
